# Effects and Molecular Mechanism of GST-Irisin on Lipolysis and Autocrine Function in 3T3-L1 Adipocytes

**DOI:** 10.1371/journal.pone.0147480

**Published:** 2016-01-22

**Authors:** Shanshan Gao, Fangmin Li, Huimin Li, Yibing Huang, Yu Liu, Yuxin Chen

**Affiliations:** 1 Key Laboratory for Molecular Enzymology and Engineering of the Ministry of Education, Jilin University, 2699 Qianjin Street, Changchun, 130012, China; 2 School of Life Sciences, Jilin University, Changchun, 130012, China; 3 National Engineering Laboratory for AIDS Vaccine, Jilin University, Changchun, 130012, China; 4 Department of Endocrinology, the Second Hospital of Jilin University, Changchun, 130041, China; GDC, GERMANY

## Abstract

Irisin, which was recently identified as a myokine and an adipokine, transforms white adipose tissue to brown adipose tissue and has increasingly caught the attention of the medical and scientific community. However, the signaling pathway of irisin and the molecular mechanisms responsible for the lipolysis effect remain unclear. In this study, we established an efficient system for the expression and purification of GST-irisin in *Escherichia coli*. The biological activity of GST-irisin was verified using the cell counting kit-8 assay and by detecting the mRNA expression of uncoupling protein 1. Our data showed that GST-irisin regulates mRNA levels of lipolysis-related genes such as adipose triglyceride lipase and hormone-sensitive lipase and proteins such as the fatty acid-binding protein 4, leading to increased secretion of glycerol and decreased lipid accumulation in 3T3-L1 adipocytes. In addition, exogenous GST-irisin can increase its autocrine function in vitro by regulating the expression of fibronectin type III domain-containing protein 5. GST-irisin could regulate glucose uptake in 3T3-L1 adipocytes. Hence, we believe that recombinant GST-irisin could promote lipolysis and its secretion in vitro and can potentially prevent obesity and related metabolic diseases.

## Introduction

Obesity, which is characterized by an excess of adipose tissue and associated with various comorbidities such as insulin resistance and type 2 diabetes, has been highlighted as an epidemic in recent decades [1, 2]. This is caused by an increase in the number and/or size of adipocytes [3]. Although the pathogenesis is extremely complicated and far from being understood, it is thought that the key component of obesity is the long-term dysregulation of energy balance, *i*.*e*., increased energy intake and/or reduced energy expenditure [2, 4]. Therefore, many studies have focused on increasing energy expenditure of adipose cells to prevent obesity.

The two main types of adipose tissue that have been well-studied are white adipose tissue (WAT) and brown adipose tissue (BAT). The main function of white adipocytes is to store energy in the form of triacylglycerols (TAGs), whereas that of brown adipocytes is to dissipate energy through the uncoupling protein 1 (UCP1)-mediated uncoupling of oxidative phosphorylation to maintain the body temperature [4, 5]. The distinctive difference in the appearance of BAT and WAT is caused by different lipid contents and the abundance of mitochondria in the adipocytes. There are more lipid droplets in WAT and more mitochondria in BAT [6]. Recently, it was discovered that certain depots of WAT, namely brite (brown in white) or beige adipocytes, could acquire a BAT phenotype when subjected to certain stimuli [7]. In fact, the “browning” of WAT is an effective anti-obesity and anti-diabetic mechanism in rodent models [8]. Lipolysis implies the breakdown of triacylglycerols and release of glycerol and fatty acids by adipose triglyceride lipase (ATGL), hormone-sensitive lipase (HSL), and monoacylglycerol lipase (MAGL) in adipocytes [6, 9–11]. Glycerol is secreted to the outside of the cell. In addition, fatty acid-binding protein 4 (FABP4), a member of the FABP family, is an important protein that can promote the solubility and transport of free fatty acids (FFAs) in adipocytes [12, 13].

Irisin, which has recently been identified as a myokine and an adipokine, transforms WAT to BAT and has increasingly caught the attention of the medical and scientific community [14–17]. Previous studies have indicated that irisin could promote the expression of UCP1 in adipocytes [14, 15, 18, 19]. However, a contrary viewpoint is that irisin has no effect on lipolysis in adipocytes [18]. Depending on the molecular mechanisms, exercise is the stimulus to produce peroxisome proliferator-activated receptor gamma coactivator-1 alpha (PGC1α), which in turn stimulates the expression of fibronectin type III domain-containing protein 5 (FNDC5), following which FNDC5 is proteolytically cleaved to release the active hormone irisin [20]. This hormone transforms WAT to BAT by the activation of the p38 mitogen-activated protein kinase (MAPK) and extracellular signal-regulated kinase (ERK)/MAPK pathways [15]. The activation of p38 MAPK is essential for PGC1α expression [21]. Treatment with irisin can result in a rapid upregulation of FNDC5 and PGC1α [14, 15, 22]. We speculate that the signaling pathway of irisin is cyclic, *i*.*e*., irisin promotes its autocrine function.

In this study, we established an efficient system for the expression and purification of GST-irisin in *Escherichia coli*. To understand the adipocyte browning process, we treated 3T3-L1 mature adipocytes with GST-irisin and examined the effects of GST-irisin on the glycerol release and the expression of genes involved in lipolysis. To further understand the molecular mechanism of irisin as a candidate for the prevention and treatment of obesity and related metabolic disorders, we examined whether GST-irisin could promote its autocrine function in mature 3T3-L1 adipocytes.

## Materials and Methods

### Plasmid construction

The protein sequence of irisin was referred to the study by Boström *et al* [14]. cDNA of human irisin was synthesized by Sangon Biotech (Shanghai, China). The PCR product and pGEX-4T-1 plasmid (Miaoling Biological Technology Co. Ltd, Wuhan, China) were cut with EcoRI and BamHI (Takara Biotech Co. Ltd, Dalian, China). The vector and fragments were joined by T4 DNA ligase (Takara Biotech Co. Ltd, Dalian, China) and transformed into *E*. *coli* BL21 cells (Novagen Co. Ltd, Madison, WI, USA). The single colonies were inoculated to Luria—Bertani medium (Dingguo Co. Ltd, Beijing, China). The sequence of pGEX-4T-1–irisin plasmid was identified by DNA sequencing (Comate Bioscience Co. Ltd, Changchun, China). The plasmid construction process is illustrated in S1 Fig.

### Expression and purification of recombinant GST-irisin in *E*. *coli* BL21

The second inoculation was accomplished from the initial bacterial culture to Luria—Bertani medium (1:100, vol/vol). After incubation at 37°C for 3 h, the optical density (OD) at 600 nm reached 0.40–0.60, following which isopropyl β-D-1-thiogalactopyranoside (IPTG) (Dingguo Co. Ltd, Beijing, China) was added until the final concentration was 1 mM. All bacterial cells were gathered after a 4-h growing period. Cells were resuspended in PBS (pH 7.3), destroyed by sonication (Ningbo Science Biotechnology Co. Ltd, Ningbo, China) and centrifuged (Eppendorf, Germany). The supernatant was gathered and loaded to the prepared Glutatathione Sepharose 4B column (General Electric Healthcare, USA). The column was washed out with the elution buffer (50 mM Tris—HCl, 10 mM reduced glutathione, pH 8.0). We have removed the elution buffer (10 mM glutathione) by dialysis before using the substance on cells. The elution buffer was loaded in the 10 kDa dialysis bag, and the dialysis bag was placed in the beaker containing 1 L PBS. Dialysis was performed with stirring at 4°C. The dialysate was refreshed in each 4 hours. The time of dialysis was 24 hours. The purity of the target protein was identified by 12% SDS-PAGE. The target protein was verified by Western Blotting using anti-irisin antibody (Phoenix Pharmaceuticals, USA). The endotoxin concentration in the target protein was measured by the chromogenic limulus method [23].

### Differentiation of 3T3-L1 preadipocytes

The 3T3-L1 preadipocytes were purchased from American Type Culture Collection (Manassas, VA, USA). Preadipocytes were cultured in Dulbecco’s modified Eagle’s medium (DMEM) containing 10% FBS (HyClone, USA) and 1% penicillin—streptomycin (Gibco, USA) and incubated at 37°C in a 5% CO_2_ incubator (Sanyo, Japan). After confluence, cells were incubated in a differentiation medium containing 0.5 mmol/L isobutylmethylxanthine (Sigma—Aldrich, USA), 0.25 mmol/L dexamethasone (Sigma—Aldrich, USA), and 10 mg/L insulin (Sigma—Aldrich, USA) in DMEM with 10% FBS. Two days later, the medium was replaced with a medium supplemented with insulin only for additional 2 days. After differentiation, human recombinant GST-irisin was added to the medium at a range of concentrations (50, 100, and 200 nM) for 2, 4, 6, and 8 days. The 3T3-L1 mature adipocytes with or without GST-irisin were processed for Oil Red O staining, RNA extraction, and western blotting.

### Cell counting kit-8 (CCK-8) assay

The 3T3-L1 preadipocytes were seeded into 96-well culture plates (ten thousand cells per well) and treated with various GST-irisin concentrations for 48 h. The cells were maintained in 10% FBS DMEM for 48 h. In total, 10 μL of CCK-8 solution (BestBio, Shanghai, China) was added to the cells and incubated for 3 h. The OD of the samples was analyzed at 450 nm using a multimode reader (infinite F200 Pro, TECAN, Switzerland), and the cell killing activity was calculated.

### RNA isolation and real-time PCR

Total RNA was extracted from the cells using an RNA extraction kit (Bioteke, Beijing, China). Quantitative and qualitative ratio metric analysis of RNA was performed. RNA integrity was confirmed using 1.5% agarose gel. Reverse transcription of 2 μg total RNA was performed using a single strand cDNA synthesis kit (Bioteke, Beijing, China). In addition, qPCR was performed in triplicate with SYBR Green I reagent (Bioteke, Beijing, China) using Applied Biosystems 7500 (Life Technologies, USA), and gene-specific primers are listed in S1 Table. The mRNA levels of the target gene were normalized to those of β-actin using the 2^−ΔΔCT^ method. Primer sequences for qPCR gene are listed in S1 Table.

### Oil Red O staining

0.5 g Oil Red O (Sigma—Aldrich, USA) was dissolved in 100 mL isopropanol (Tianjin, China), following which 6 mL of this solution was mixed with 4 mL water. The 3T3-L1 mature adipocytes were washed once with PBS and fixed with 3.7% formaldehyde (Sigma—Aldrich, USA) at room temperature for 20 min and then incubated with the staining solution for 1 h. The cells were washed twice with water. Pictures were taken using an Olympus microscope (Tokyo, Japan). Following this, 450 μL isopropanol was added, and the cells were kept for 5 min. Next, 200 μL of the eluate from each well was transferred to a 96-well plate, and the absorbance values at a 492-nm wavelength were measured using a multimode reader (infinite F200 Pro, TECAN, Switzerland).

### Glycerol release

The treated cells were washed once with PBS and were then incubated with DMEM (no phenol red) (Gibco, USA) with or without GST-irisin for 4 h. Samples of the media were collected and assayed for glycerol levels using a glycerol assay kit (Nanjing Jiancheng Bioengineering Institute, Nanjing, China).

### Irisin release

The treated cells were washed one to three times with PBS and were then incubated with DMEM containing 10% FBS without GST-irisin for 4 h. Samples of the media were collected and measured for irisin concentrations using a commercial ELISA kit (catalog number: EK-067-29, Phoenix Pharmaceuticals, USA).

### Detection of glucose concentrations

Every 2 days, fresh media with respective GST-irisin concentration was administered to cells. Samples of the medium were collected and measured for glucose concentrations using a glucose detection kit (BestBio, Shanghai, China).

### Western blotting

The treated cells were washed with ice-cold PBS and solubilized in a RIPA lysis buffer (Beyotime, Shanghai, China) containing Protease Inhibitor Cocktail (Sigma-Aldrich, USA). After incubating on ice for 2 h, the insoluble materials were removed by centrifugation at 12 000 rpm for 15 min at 4°C. Accurate protein concentrations were determined using the Pierce^™^ BCA Protein Assay Kit (Thermo, USA). 25 μg of protein were loaded into each well of 12% SDS-PAGE. Proteins were transferred to PVDF membranes (Millipore, USA), blocked for 2.5 h with 5% Difco^™^ Skim Milk (BD, USA) in PBS at room temperature, and blotted with the indicated primary antibodies (FABP4 (catalog number: ab92501), ATGL (catalog number: ab109251), Abcam, England; FNDC5 (catalog number: bs-8486R), BIOSS, Beijing, China) overnight at 4°C at 1:5000 dilution. Anti-β-actin antibody (catalog number: bs-0061R, BIOSS, Beijing, China) was used at 1:1000 dilution. After washing with PBS with Tween-20, the membranes were incubated with horseradish peroxidase (HRP)-conjugated secondary antibodies for 2 h at room temperature. The blots were detected using enhanced chemiluminescence (ECL) (Millipore, USA) method.

### Statistical analysis

The data were presented as mean ± standard deviation (SD) of at least three independent experiments and were analyzed using Student’s *t*-tests and one-way ANOVA with the SPSS 19.0 software program. *P* < 0.05 was considered as statistically significant.

## Results

### Expression and purification of recombinant GST-irisin from *E*. *coli* BL21

**Fig 1:**
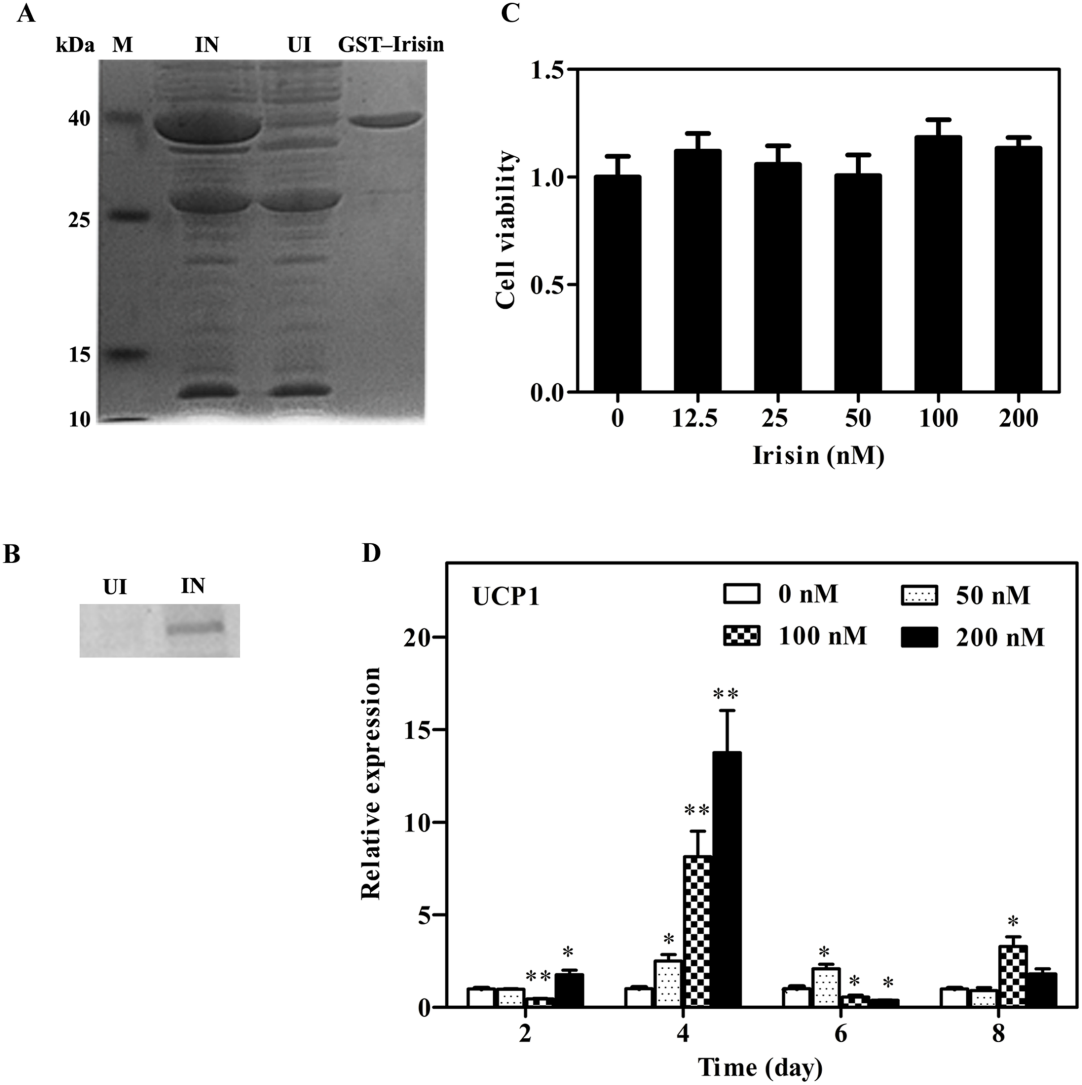
A: SDS-PAGE analysis of pGEX-4T-1–irisin expression and purification. M: low molecular weight marker (kDa); IN: induced culture; UI: un-induced culture; GST-Irisin: the purified eluent. B: Western Blotting of GST-irisin. IN: induced culture; UI: un-induced culture. C: Effect of GST-irisin on 3T3-L1 preadipocytes. 3T3-L1 preadipocytes were treated with various GST-irisin concentrations (0, 12.5, 25, 50, 100, and 200 nM), and the cell viability was assessed using the CCK-8 assay. D: qPCR of UCP1 of 3T3-L1-derived adipocytes. 3T3-L1 mature adipocytes were cultured with various GST-irisin concentrations (0, 50, 100, and 200 nM). The expression of UCP1 mRNA was determined by qPCR. Values are expressed as the mean ± SD of three independent experiments. *P < 0.05 versus control; **P < 0.01 versus control.

To achieve high-yield expression of the irisin gene in an *E*. *coli* expression system, an optimized irisin cDNA coding sequence for *E*. *coli* codon usage was designed and constructed. Glutathione S-transferase (GST)-tag is commonly used as a fusion tag to enhance the expression and solubility of proteins. To investigate the effect of GST-tag on the expression of GST-irisin, the irisin gene sequence was inserted into the pGEX-4T-1 plasmid to build pGEX-4T-1–irisin. The plasmid was transformed into *E*. *coli* BL21, and the expression of the GST-irisin fusion protein was induced by IPTG. SDS-PAGE showed that the fusion protein was expressed in the soluble portion. The weight of GST-irisin was found to be approximately 37 kDa, as indicated by the arrow in Fig 1A. These results indicate that GST-tag is suitable for the expression and rapid purification of GST-irisin and should be amenable to high-throughput applications. The verified result of GST-irisin was shown in Fig 1B. The level of endotoxin in the target protein is less than 5 Eu/ml, and the data is far less than the minimum value of the lipopolysaccharide that stimulated lipolysis in adipocytes [24].

### Effects of different GST-irisin concentrations on 3T3-L1 preadipocytes

The CCK-8 reagent can be used for simple and accurate analysis of cell proliferation and toxicity. CCK-8 assays were performed with 3T3-L1 cells to evaluate the effect of GST-irisin on cell proliferation. There was no significant difference between the treated group and the control group for 48 h (*P* > 0.05). In addition, the proliferation ability between the treated groups (12.5, 25, 50, 100, and 200 nM) showed no significant difference (*P* > 0.05) (Fig 1C). No effect on 3T3-L1 cell viability was observed at these GST-irisin concentrations.

### Verification of irisin protein activity

To verify the activity of GST-irisin obtained by prokaryotic expression and to investigate the browning effect on adipocytes, 3T3-L1-derived adipocytes as a cellular model were treated with or without GST-irisin to evaluate the mRNA expression of UCP1 by qPCR. The results showed that the treatment of the 3T3-L1-derived adipocytes with GST-irisin (50, 100, and 200 nM) significantly upregulated the brown cell marker UCP1 on the fourth dosing day for all tested concentrations (Fig 1D). This effect decreased dramatically when the 3T3-L1-derived adipocytes were treated with GST-irisin for a longer time. The primer sequences for qPCR are shown in S1 Table.

### Effect of GST-irisin on lipid accumulation in 3T3-L1 mature adipose cells

**Fig 2:**
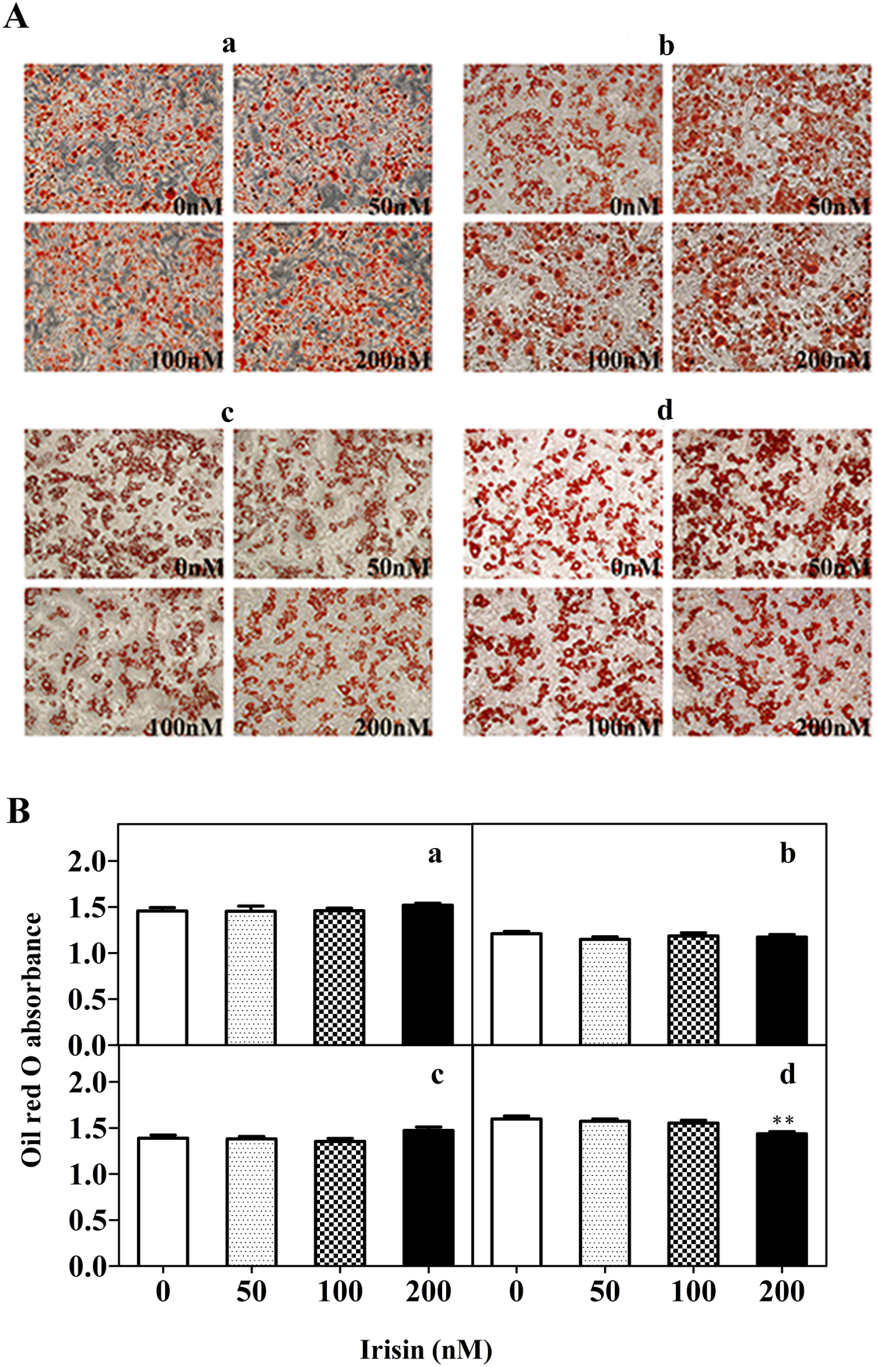
Fat droplets in control and treated groups. 3T3-L1 mature adipocytes were cultured with various GST-irisin concentrations (0, 50, 100, and 200 nM) for 2 (a), 4 (b), 6 (c) and 8 (d) days. A: Oil Red O staining (200×). B: OD values of isopropanol extraction. Values are expressed as the mean ± SD of three independent experiments. **P < 0.01 versus control.

Oil Red O dye can strongly combine with triglycerides. Oil Red O staining was performed to quantitatively examine the effect of GST-irisin on lipid droplet accumulation by spectrophotograpy. After complete differentiation, mature adipocytes were treated with GST-irisin for 2, 4, 6, and 8 days. The photographs indicate that almost all 3T3-L1 cells displayed a mature adipocyte phenotype and there was no difference between the control and treated groups (Fig 2A). However, isopropanol extraction of Oil Red O revealed that increasing GST-irisin concentrations had no significant effect on lipid accumulation in cells treated for 2, 4, and 6 days, whereas there was a decrease in lipid accumulation in cells treated with GST-irisin for 8 days. The OD values of the 200 nM groups were statistically different compared with those of the control group (*P* < 0.01) (Fig 2B).

### Effect of GST-irisin on lipolysis in 3T3-L1 mature adipose cells

**Fig 3:**
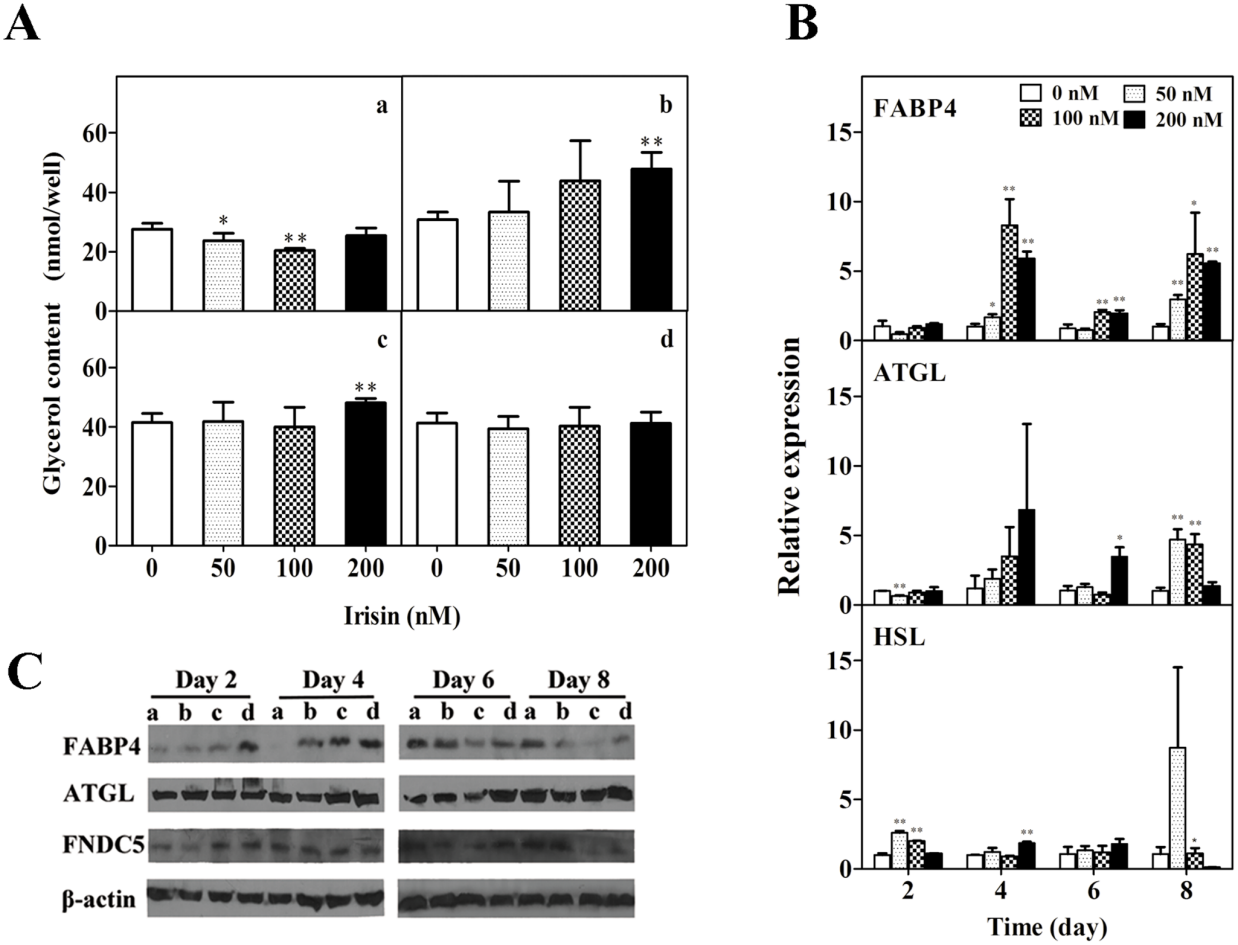
Effect of GST-irisin on lipolysis in 3T3-L1 mature adipocytes. A: Effect of GST-irisin on the release of glycerol in 3T3-L1 mature adipocytes. The adipocytes were cultured with various GST-irisin concentrations (0, 50, 100, and 200 nM) for 2 (a), 4 (b), 6 (c) and 8 (d) days. B: The mRNA expression of FABP4, ATGL, and HSL was determined by qPCR. C: The protein levels of FABP4, ATGL, FNDC5, and β-actin were determined by western blotting. The adipocytes were cultured with various GST-irisin concentrations, and the concentration was 0 (a), 50 (b), 100 (c), and 200 (d) nM. Values are expressed as the mean ± SD of three independent experiments. *P < 0.05 versus control; **P < 0.01 versus control.

To determine whether irisin plays a role in lipolysis in adipocytes, differentiated 3T3-L1 adipocytes were treated with different GST-irisin concentrations. The glycerol content in the culture medium had an obvious linear relationship for 4 and 6 days; however, this phenomenon disappeared as the culture time extended. This may be because the cells adapted to GST-irisin stimulation, *i*.*e*., irisin resistance (Fig 3A). Treatments with GST-irisin over a range of concentrations (50, 100, and 200 nM) significantly increased the mRNA level of FABP4 and ATGL for 4, 6, and 8 days. GST-irisin slightly increased the mRNA level of HSL for 2 days but had no significant impact as the culture time extended (Fig 3B). GST-irisin increased the protein level of FABP4 for 2 and 4 days but decreased it for 6 and 8 days. GST-irisin increased the protein expression of ATGL for 4 and 6 days but had no significant effect on the second and eighth day (Fig 3C).

### Effect of GST-irisin on autocrine function in 3T3-L1 mature adipose cells

**Fig 4:**
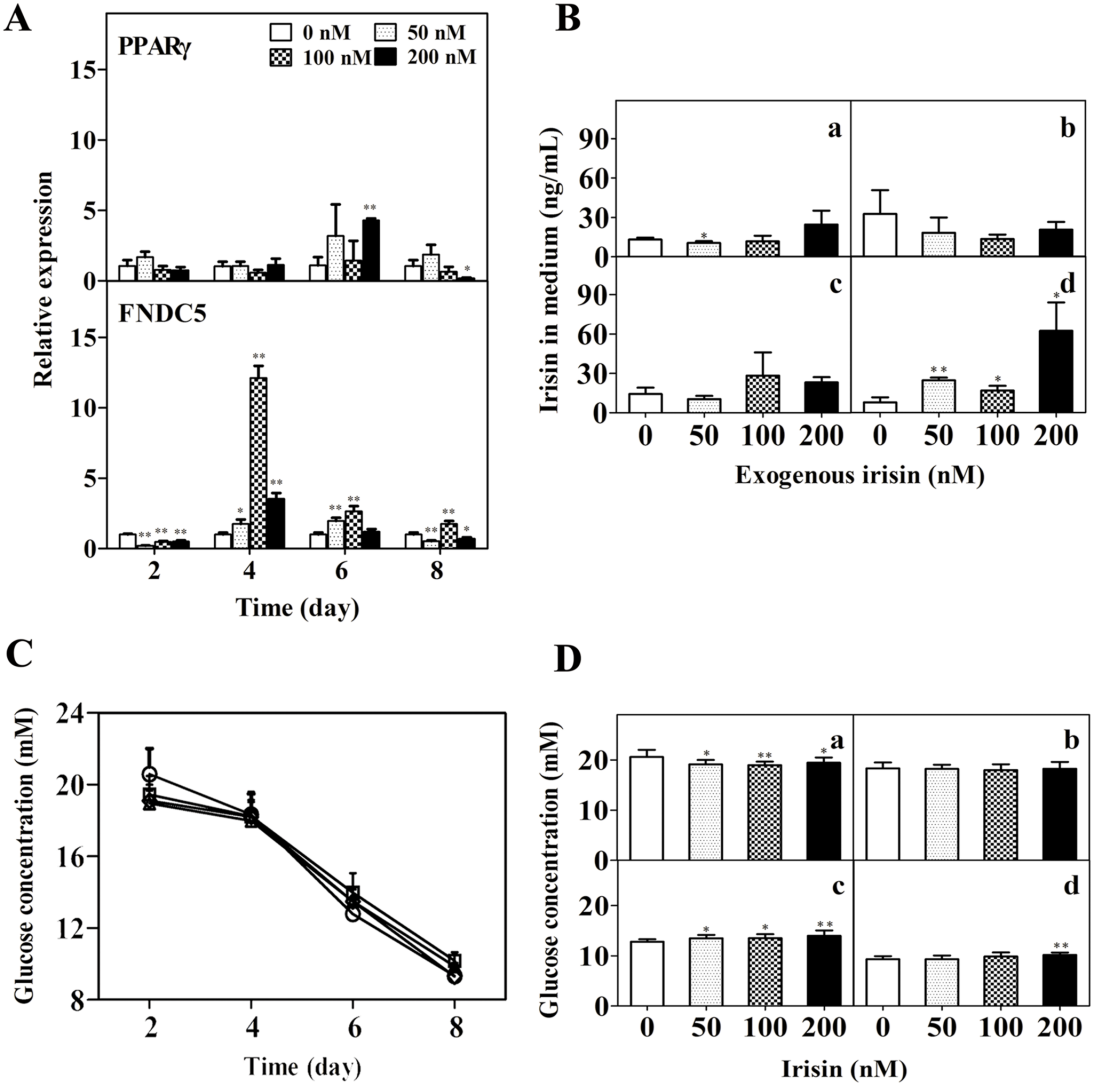
Effect of GST-irisin on the autocrine function and glucose concentration in 3T3-L1 mature adipose cells. A: Effect of GST-irisin on the genes upstream of itself (PPARγ and FNDC5) in 3T3-L1 mature adipocytes. The mRNA expression was determined by qPCR. B: The mature adipocytes were cultured with various GST-irisin concentrations (0, 50, 100, and 200 nM) for 2 (a), 4 (b), 6 (c), and 8 (d) days. The irisin concentrations in the culture media in 3T3-L1 mature adipocytes were determined by ELISA. C: The GST-irisin concentration was 0 (white circles), 50 (white diamond), 100 (white triangle), and 200 (white squares) nM. D: The adipocytes were cultured with GST-irisin for 2 (a), 4 (b), 6 (c), and 8 (d) days. Values are expressed as the mean ± SD of 10 independent experiments. *P < 0.05 versus control; **P < 0.01 versus control.

Treatments with GST-irisin over a range of concentrations significantly increased mRNA levels of FNDC5 for 4, 6, and 8 days but had no significant influence on peroxisome proliferator-activated receptor γ (PPARγ) except the results of 200 nM in 6 and 8 days (Fig 4A). GST-irisin increased the protein level of FNDC5 for 2 days and had no significant influence for 4 and 6 days, while it decreased the level on the eighth day (Fig 3C). Irisin is an adipokine; therefore, irisin concentrations in the culture media in 3T3-L1 mature adipocytes were determined by ELISA. To eliminate the influence of exogenous GST-irisin, the adipocytes treated with or without recombinant GST-irisin were washed with PBS and then exchanged in DMEM media containing 10% FBS without GST-irisin for 4 h. There was roughly no difference between GST-irisin concentrations in the culture media of the treated and control groups for 2, 4, and 6 days. Exogenous GST-irisin promoted its autocrine function in the culture media of the mature 3T3-L1 adipocytes for 8 days, and this phenomenon was directly related to GST-irisin concentrations (Fig 4B).

### Effect of GST-irisin on glucose concentrations in the cell culture

Because of the close correlation between glucose metabolism and lipid metabolism, we determined whether GST-irisin affects glucose concentrations in the culture media. Glucose concentrations dramatically decreased as the culture time increased (Fig 4C). Glucose concentrations of the dosing groups on the second day were lower than those of the control group; in contrast, the concentrations became higher than those of the control group on the sixth day. The concentration in the 200 nM group was also higher than that of the control group on the eighth day (Fig 4D).

## Discussion

Irisin, a novel myokine/adipokine, promotes the “browning” of adipocytes in vitro and in vivo. The discovery of irisin has provided the opportunity to further study the role of adipocytes in obesity and obesity-related metabolic disorders [20, 25–27]. However, the signaling pathway of irisin and the molecular mechanisms underlying lipolysis are unclear.

Prokaryotic expression systems are extensively utilized for heterogenic protein expression [28]. In our study, the prokaryotic expression plasmid containing optimal codon usage of the irisin coding sequence was successfully constructed and purified. The recombinant GST-irisin protein from *E*. *coli* with full biological functions was generated with a good harvest. This approach provides a solid foundation for the future industrial production of irisin. Human recombinant GST-irisin protein expressed in *E*. *coli* showed an approximately 37-kDa band. The reason is that the size of GST is 26 kDa and the length of irisin consists of 112 amino acids [14, 29]. We tried to cleave the GST-tag of recombinant protein with thrombin several times. It was quite interesting that the truncated protein after cleavage precipitated in PBS solution each time, hence the recombinant protein without GST-tag was not used for *in vitro* studies. Thus, we made use of the recombinant protein with GST-tag for convenience and it was renamed “GST-irisin” in our paper. As reported previously, irisin can form dimmers [30], which may have contributed to precipitate formation. No effect on 3T3-L1 cell viability was detected at GST-irisin concentrations in this study, which is consistent with the findings of Wang *et al*. [18]. However, previous studies have reported that irisin can promote cell proliferation in human umbilical vein endothelial cells [31] and mouse H19-7HN cells [32] but significantly decrease cell number, migration, and viability in malignant MDA-MB-231 cells [33].

Since the amino acid homologies are 100% between mouse and human, mouse adipocytes were used to evaluate the effects of GST-irisin in this study. Our results demonstrated that GST-irisin could regulate lipolysis. GST-irisin increased the expression of lipolysis-related genes such as HSL, ATGL, and FABP4 in the 3T3-L1 adipocytes over a specific time range. An increase in the glycerol content in the treated media was also detected for 10 and 12 days (data not shown), indicating that GST-irisin may enhance lipolysis but requires a long reaction time in adipocytes. In a previous study, Wang *et al*. proposed that increasing irisin concentrations had no significant influence on lipolysis in adipocytes [18], which is not consistent with the data in our study. The reason may be because the 3T3-L1 adipocytes were treated with GST-irisin obtained from prokaryotic expression for 8 days in our study, whereas in the study by Wang *et al*., the length of experimental time was 48 h and irisin was produced by *Pichia pastoris* [18]. Zhang *et al*. found that irisin is inversely associated with intrahepatic triglyceride contents in obese adults [34], which was consistent with our data. A longer time and a higher concentration of irisin may be required to promote lipid degradation. Some investigators have inferred that FABP4 is involved in the regulation of glucolipid metabolism in relation to inflammatory and metabolic processes in target cells, particularly adipocytes and macrophages [35]. Therefore, the effects of GST-irisin on mRNA and protein levels of FABP4 in our study suggest the importance of irisin in glucose and lipid metabolism.

Several studies have reported that irisin promotes the mRNA expression of FNDC5 [14, 15, 22, 36]. The results showed that GST-irisin could reduce the mRNA expression of FNDC5 in the 3T3-L1 adipocytes over a short period (2 days) and promote the expression in 4, 6 days, respectively (Fig 4A). There is no obvious linear relationship of the dose-dependence and the time-dependence. Colaianni G *et al*. found that low-dose irisin regulated osteoblast gene expression, but did not cause a browning response *in vivo* [36], which showing the complexity of the regulation of irisin. Because FNDC5 is the precursor of irisin, we speculate that irisin promotes its own expression. The results of ELISA on the eighth day verified the hypothesis that GST-irisin can promote its autocrine function in 3T3-L1 adipocytes (Fig 4B). Actually, the irisin detected in Fig 4B contains bovine irisin in culture medium and the secreted irisin of mature fat cells. We tried to detect the single secreted irisin of mature fat cells using DMEM without FBS for 4 hours. Unfortunately, we failed. The reason may be due to that cells were hungry in serum-free medium and the concentration of the secreted irisin of mature fat cells was too low and it was undetectable by the ELISA kits. In addition, glucose concentrations in the media were determined on account of the close correlation with glycolipid metabolism. The results showed that GST-irisin could increase glucose uptake in the 3T3-L1 adipocytes in 2 days and reduce it in 6 days (Fig 4D). This may be because the cells adapted to GST-irisin stimulation, *i*.*e*., irisin resistance in glucose metabolism. We speculate that GST-irisin may need a relatively long time to participate in glucose metabolism, however, Vaughan *et al*. showed that irisin increased both glycolytic and mitochondrial metabolisms as fast as 1 hour following a much lower treatment (5 nM) in skeletal muscle cell [37], which is not consistent with the data in this study. The reason may be attributed to that glucose metabolism is different between skeletal muscle cells and adipocytes. These conclusions may pave a way for further glucose metabolism studies in vivo.

**Fig 5:**
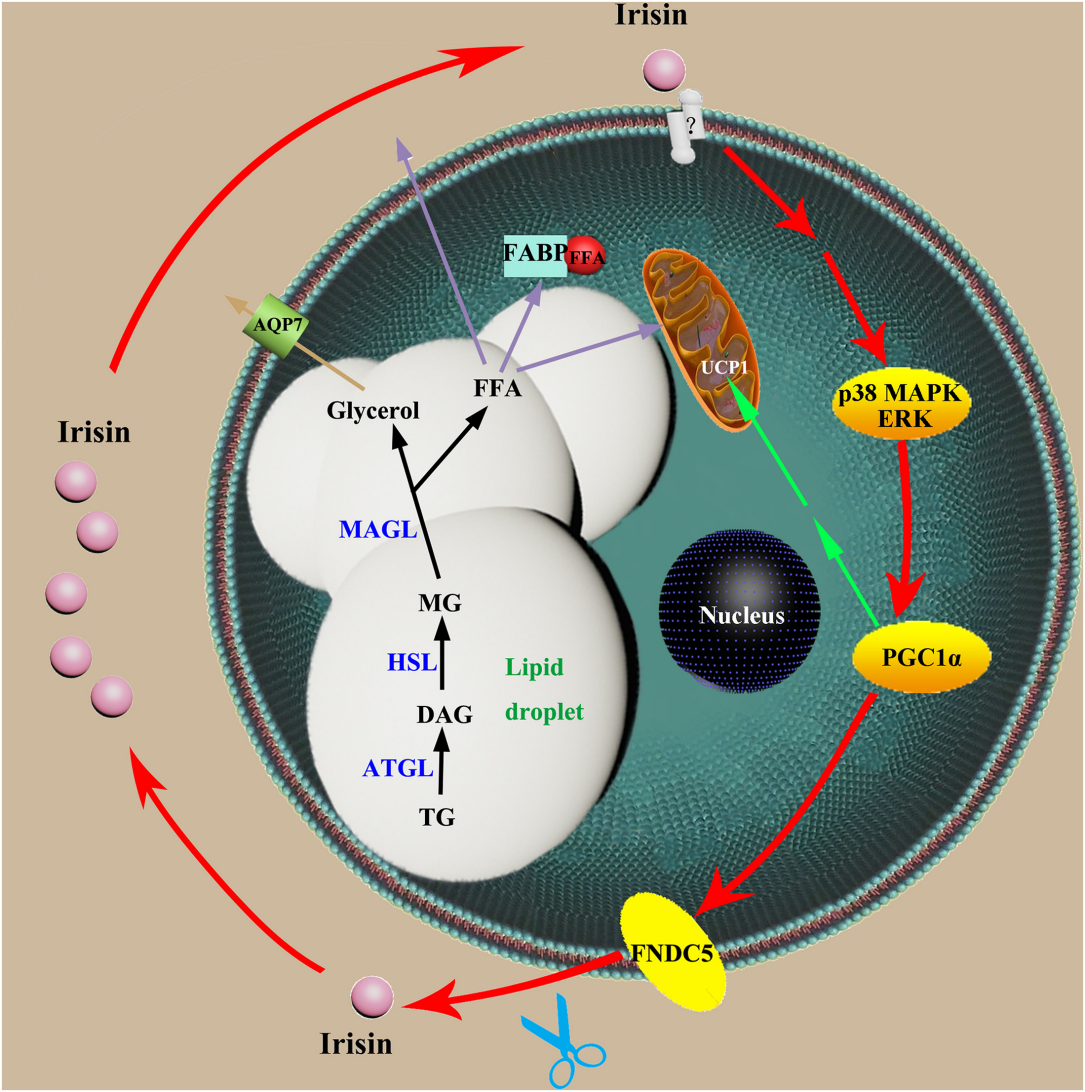
The proposed molecular mechanism of irisin in adipocytes. AQP7: aquaporin 7, ATGL: adipose triglyceride lipase, DAG: diacylglycerol, ERK: extracellular signal-regulated kinase, FABP: fatty acid-binding protein, FFA: free fatty acid, FNDC5: Fibronectin Type III Domain Containing 5, HSL: hormone-sensitive lipase, MAGL: monoacylglycerol lipase, MAPK: mitogen-activated protein kinase, MG: monoacylglycerol, PGC1α: peroxisome proliferator-activated receptor gamma coactivator 1-alpha, TG: triglyceride, UCP1: uncoupling protein 1.

On the basis of our data, we proposed the molecular mechanisms underlying the self-promoted cycle of irisin in adipocytes. As shown in Fig 5, extracellular GST-irisin combines with its unknown receptors [14] and stimulates the p38 MAPK and ERK/MAPK signaling [15]. The signal upregulates PGC1α and then increases the expression of FNDC5 [14, 15, 20–22, 38]. FNDC5 is proteolytically cleaved to release the active hormone irisin [14]. The signaling pathway of irisin may be cyclic, i.e., irisin may promote its autocrine function. In addition, irisin has effects on the lipolysis-related molecules such as HSL, ATGL, and FABP4 resulting in increased glycerol release and decreased lipid accumulation in adipocytes.

In summary, the data revealed that recombinant GST-irisin enhanced lipolysis by regulating the expression of ATGL, HSL, and FABP4 and promoted its own secretion in vitro by regulating the expression of FNDC5. Based on our results, we proposed molecular mechanisms underlying the self-promoted cycle of irisin in adipocytes. In addition, GST-irisin could regulate glucose uptake in 3T3-L1 adipocytes. These findings provide preliminary experimental evidence for the potential use of recombinant irisin for the treatment of obesity and obesity-related metabolic disorders.

## Supporting Information

S1 Fig Map of pGEX-4T-1 and nucleotide sequence of synthesized irisin. (TIF)

S1 Table Primer sequences for qPCR (DOC)
