# Supplementary figures and images for: Effects and Molecular Mechanism of GST-Irisin on Lipolysis and Autocrine Function in 3T3-L1 Adipocytes
Source: PLoS One. 2016 Jan 22;11(1):e0147480. doi: 10.1371/journal.pone.0147480 (PMC4723061; doi:10.1371/journal.pone.0147480)

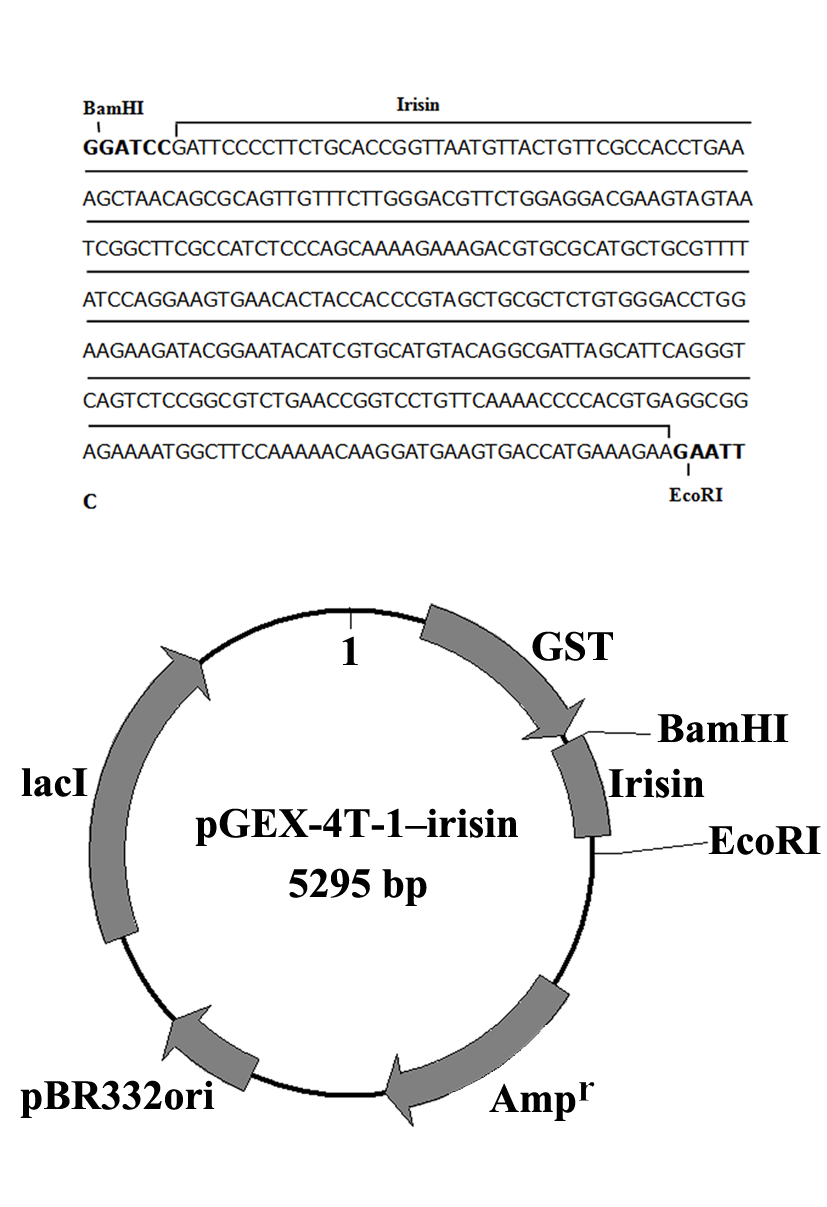

Supplement: S1 Fig — (TIF) [file pone.0147480.s001.tif]
