# Supplementary material for: Effects and Molecular Mechanism of GST-Irisin on Lipolysis and Autocrine Function in 3T3-L1 Adipocytes
Source: PLoS One. 2016 Jan 22;11(1):e0147480. doi: 10.1371/journal.pone.0147480 (PMC4723061; doi:10.1371/journal.pone.0147480)
Supplement: S1 Table — (DOC) [file pone.0147480.s002.doc]

**Table S**1. Primer sequences for qPCR

| ***Gene*** | ***Forward sequence (5′–3′)*** | ***Reverse sequence (5′–3′)*** |
| --- | --- | --- |
| β-actin | TCCATCATGAAGTGTGACGT | GAGCAATGATCTTGATCTTCAT |
| UCP1 | AGGCTTCCAGTACCATTAGGT | CTGAGTGAGGCAAAGCTGATTT |
| FABP4 | TCACCTGGAAGACAGCTCCT | AATCCCCATTTACGCTGATG |
| ATGL | GGTGCCAACATTATTGAGGTG | AAACACGAGTCAGGGAGATGC |
| HSL | TCGGGGAGCACTACAAACG | CACGCAACTCTGGGTCTATGG |
| PPARγ | TTTTCCGAAGAACCATCCGATT | ATGGCATTGTGAGACATCCCC |
| FNDC5 | CACGCGAGGCTGAAAAGATG | CCTTGTTGTTATTGGGCTCGTT |
